# Burden of fine air pollution on mortality in the desert climate of Kuwait

**DOI:** 10.1038/s41370-023-00565-7

**Published:** 2023-06-15

**Authors:** Barrak Alahmad, Jing Li, Souzana Achilleos, Fahd Al-Mulla, Ali Al-Hemoud, Petros Koutrakis

**Affiliations:** 1 Department of Environmental Health, Harvard T.H. Chan School of Public Health, Boston, MA USA; 2 Environmental & Occupational Health Department, College of Public Health, Kuwait University, Kuwait City, Kuwait; 3 Dasman Diabetes Institute, Kuwait City, Kuwait; 4 Institute of Child and Adolescent Health, School of Public Health, Peking University, Beijing, China; 5 Department of Primary Care & Population Health, University of Nicosia Medical School, Nicosia, Cyprus; 6 Environment & Life Sciences Research Center, Kuwait Institute for Scientific Research, Kuwait City, Kuwait

**Keywords:** Middle East, Arabian Peninsula, PM2.5, Deaths, Gulf

## Abstract

**Background:**

Middle Eastern desert countries like Kuwait are known for intense dust storms and enormous petrochemical industries affecting ambient air pollution. However, local health authorities have not been able to assess the health impacts of air pollution due to limited monitoring networks and a lack of historical exposure data.

**Objective:**

To assess the burden of PM_2.5_ on mortality in the understudied dusty environment of Kuwait.

**Methods:**

We analyzed the acute impact of fine particulate matter (PM_2.5_) on daily mortality in Kuwait between 2001 and 2016. To do so, we used spatiotemporally resolved estimates of PM_2.5_ in the region. Our analysis explored factors such as cause of death, sex, age, and nationality. We fitted quasi-Poisson time-series regression for lagged PM_2.5_ adjusted for time trend, seasonality, day of the week, temperature, and relative humidity.

**Results:**

There was a total of 70,321 deaths during the study period of 16 years. The average urban PM_2.5_ was estimated to be 46.2 ± 19.8 µg/m^3^. A 10 µg/m^3^ increase in a 3-day moving average of urban PM_2.5_ was associated with 1.19% (95% CI: 0.59, 1.80%) increase in all-cause mortality. For a 10 µg/m^3^ reduction in annual PM_2.5_ concentrations, a total of 52.3 (95% CI: 25.7, 79.1) deaths each year could be averted in Kuwait. That is, 28.6 (95% CI: 10.3, 47.0) Kuwaitis, 23.9 (95% CI: 6.4, 41.5) non-Kuwaitis, 9.4 (95% CI: 1.2, 17.8) children, and 20.9 (95% CI: 4.3, 37.6) elderly deaths each year.

**Impact Statement:**

- The overwhelming prevalence of devastating dust storms and enormous petrochemical industries in the Gulf and the Middle East has intensified the urgency to address air pollution and its detrimental health effects. Alarmingly, the region’s epidemiological research lags behind, hindered by a paucity of ground monitoring networks and historical exposure data. In response, we are harnessing the power of big data to generate predictive models of air pollution across time and space, providing crucial insights into the mortality burden associated with air pollution in this under-researched yet critically impacted area.

## Background

The Middle East and Gulf region are commonly affected by severe, and rather dramatic, dust storms [1]. Desert countries in this region experience varying frequencies and magnitudes of dust storms depending on their geographical location and time of year [2]. For example, in Kuwait, more than 270 tons of dust per km^2^ per year get dumped in Kuwait City, making it the highest in the world [3]. Additionally, Kuwait is also one of the largest oil producers in the world with enormous petrochemical industries that can further deteriorate ambient air quality in the area [4]. Between 2017 to 2019 in Kuwait, fine particulate matter (PM_2.5_) levels exceeded the daily World Health Organization (WHO) guideline of 25 µg/m^3^ in 74% of the days in Kuwait City and 90% of the days in a location near petrochemical facilities [5].

The detrimental health effects of air pollution have been extensively documented across various regions, including the Americas, Europe, and Asia [6–8]. However, the epidemiology of air pollution remains underdeveloped in certain areas. Consequently, local health authorities find it very difficult to come up with air pollution standards that accurately reflect local health vulnerabilities. Data scarcity is a primary contributor to these gaps, as limited ground monitoring networks have led to temporal gaps in historical exposure data. Furthermore, the available field measurements often exhibit poor spatial resolution and questionable accuracy, particularly during extreme dust events when ill-equipped devices can easily become overwhelmed [5, 9].

Fortunately, the existence of large satellite data allowed for sophisticated modern-day models to predict air pollution concentrations over time and space [10, 11]. The new spatio-temporal air pollution models provide a unique opportunity to perform environmental health analyses in under-studied areas that are extremely polluted [12, 13].

Previous pollution and health studies in Kuwait relied on large particulate matter (PM_10_) [14], low visibility [15] and seasonal dust trends [16] as a proxies for air pollution, or respiratory hospital admissions as an upstream health outcome [17, 18]. In this paper, we employed a previously developed model that estimated historically resolved fine particulate matter in the dusty environment of Kuwait, allowing us to more comprehensively assess its impact on mortality. This paper delves into the effects of PM_2.5_ on various causes of death and accounts for diverse population characteristics such as sex, age, and nationality, providing a more thorough understanding of the health burden for decision-makers.

## Methods

### Exposure data

We used previously predicted PM_2.5_ concentrations for the study period from 2001 to 2016; detailed description of the model can be found elsewhere [12]. Prediction outputs were obtained from a hybrid PM_2.5_ prediction model that combined machine learning methods and generalized additive mixed models. The model provided high spatial (1 × 1 km) and temporal (daily) resolution for all areas in Iraq and Kuwait. The R-squared value was 0.71. The model utilized visibility, satellite retrievals of aerosol optic depth, land use data, and ground-based observations. We averaged the daily PM_2.5_ levels from all urban pixels in Kuwait without barren desert lands. The exposure data is available from the authors upon reasonable request [12, 13].

For the same study period, we also collected 24-hour average temperature (°C) and relative humidity (%) from the Meteorological Department of the Directorate General of Civil Aviation in Kuwait.

### Mortality data

We extracted 16 years (from January 1st, 2001, to December 31st, 2016) of daily mortality data from the administrative death registry at the National Centre for Health Information, Department of Vital Statistics, Ministry of Health, Kuwait. Only deaths inside Kuwait were included. Diagnoses according to the International Classification of Disease version 10 (ICD-10) codes were used to differentiate causes of death. We analyzed all-cause non-accidental (ICD-10: A00-R99), cardiovascular (ICD-10: I00-I99), and respiratory (ICD-10: J00-J99) mortality. We further stratified the daily mortality by three age categories; children (<15 years), adults (15–65 years), and the elderly (65+ years). Other information available on death certificates include sex (male, female) and nationality (Kuwaiti, non-Kuwaiti).

### Statistical analysis

We fitted quasi-Poisson timeseries regression to assess the association between daily counts of mortality and daily PM_2.5_ exposure, to adjust for overdispersion. Modelling choices were based on best fit determined by an extended version of the Akaike Information Criterion (AIC) for quasi-likelihood (qAIC) [19].

First, we assessed linearity of PM_2.5_ using penalized splines from generalized additive models (GAM) [20]. We explored penalized splines for all mortality outcomes to assess all exposure-response relationships. Second, we investigated the effects of PM_2.5_ using two different lag structures: distributed lag models (DLM) and moving averages. Our objective was to explore and compare the temporal patterns of the lagged associations between PM_2.5_ and mortality. In the case of distributed lag models, we fitted linear terms for each lag day and then calculated the sum of the coefficients to capture the overall effect. We considered both 3-day and 5-day lags to evaluate the potential impact of short-term exposure to PM_2.5_. For the moving average approach, we examined the associations using various time windows, specifically 2-day, 3-day, 5-day, and 7-day moving averages. This allowed us to assess the consistency and robustness of the observed associations between PM_2.5_ and the outcomes across different averaging periods. Third, we fitted natural splines with 4 to 7 degrees of freedom per year to adjust for seasonality and long-term trend. Fourth, we adjusted for temperature using distributed lag nonlinear models (DLNM), a cross-basis where temperature-response and lag-response are modelled simultaneously [21]. We specified the temperature-response dimension using natural splines with 3 degrees of freedom. In terms of the lag-response dimension, we explored both 7-day and 14-day lag periods, modeling these using natural splines with 2 to 3 degrees of freedom, equally spaced in the log scale. Finally, we adjusted for day of the week (categorical variable) and relative humidity (penalized splines).

The final model with the best fit included 3-day moving average for the PM_2.5_, time control with 5 degrees of freedom per year, DLNM for temperature with 7 days lag and natural spline with 2 degrees of freedom spaced equally in the log scale of lag.

The effect estimates were reported as percentage increase in mortality for every 10 μg/m^3^ increase in PM_2.5_:

$$(exp(\beta \times 10) - 1) \times 100{{{{{{{\mathrm{\% }}}}}}}}$$

Where β is the regression coefficient of PM_2.5_

The burden of mortality was reported as number of preventable deaths each year for every 10 μg/m^3^ reduction in PM_2.5_ [22, 23]:

$$(exp(\beta \times 10) - 1) \times Total\;number\;of\;deaths/Number\;of\;years$$

We employed a two-sample z-test to statistically assess significant differences in the effect estimates across categories within each subgroup. This analysis was based on the point estimate and standard error (se). For example, we conducted this assessment for comparisons between Kuwaitis vs. non-Kuwaitis, females vs. males, as well as adults vs. elderly individuals [24].

$$Z = \frac{{\beta _1 - \beta _2}}{{\sqrt {se(\beta _1)^2 + se(\beta _2)^2} }}$$

All analyses were carried out using R statistical software version 4.0.3 (R Foundation for Statistical Computing, Vienna, Austria).

## Results

A total of 70,321 deaths were analyzed during a 16-year time period (2001–2016) (Table 1). The majority of deaths were from cardiovascular causes (47.9%), with an average of 5.8 deaths every day. Only 7.8% of deaths in Kuwait were from respiratory causes. Males had higher death rates than females (7.2 vs. 4.9 deaths per day). On average, about five adults and five elders died from all-causes every day. Children daily deaths were much less, almost one death per day. Of the total deaths inside Kuwait, 53.2% were Kuwaitis and 46.8% were non-Kuwaitis.

**Table 1:** Summary statistics of mortality, fine air pollution and meteorological variables in Kuwait for the period from 2001–2016.

	*N* (%)	Mean	SD	Median	Range
Overall Deaths (per day)	70,321 (100%)	12.0	(4.6)	12.0	[0, 45.0]
*Cardiovascular*	33,649 (47.9%)	5.8	(2.8)	5.0	[0, 22.0]
*Respiratory*	5512 (7.8%)	0.9	(1.1)	1.0	[0, 8.00]
*Males*	41,837 (59.5%)	7.2	(3.2)	7.0	[0, 26.0]
*Females*	28,484 (40.5%)	4.9	(2.6)	5.0	[0, 21.0]
*Children (<15)*	8035 (11.4%)	1.4	(1.3)	1.0	[0, 10.0]
*Adults (15–65)*	31,738 (45.1%)	5.4	(2.7)	5.0	[0, 28.0]
*Elderly (65*+*)*	30,548 (43.4%)	5.2	(2.7)	5.0	[0, 23.0]
*Kuwaitis*	37,395 (53.2%)	6.4	(3.0)	6.0	[0, 28.0]
*Non-Kuwaitis*	32,926 (46.8%)	5.6	(2.8)	5.0	[0, 26.0]
Temperature (°C)	-	27.1	(9.8)	28.1	[5.19, 44.0]
Relative Humidity (%)	-	34.3	(21.4)	28.7	[5.20, 99.0]
PM_2.5_ (µg/m^3^)	-	46.2	(19.8)	42.7	[10.4, 746]

The mean annual predicted urban PM_2.5_ was 46.2 µg/m^3^ (standard deviation [SD] = 19.8). The 24-hour average temperature and relative humidity were 27.1 °C (SD = 9.8) and 34% (SD = 21.4%), respectively.

The exact shapes of the exposure-response relationships are shown in Fig. S1. While there might be some deviations from linearity in some curves, the low effective degrees of freedom from fitted penalized splines (<3) indicated that the smooth term adds limited complexity to the model beyond a linear fit.

**Fig. 1:**
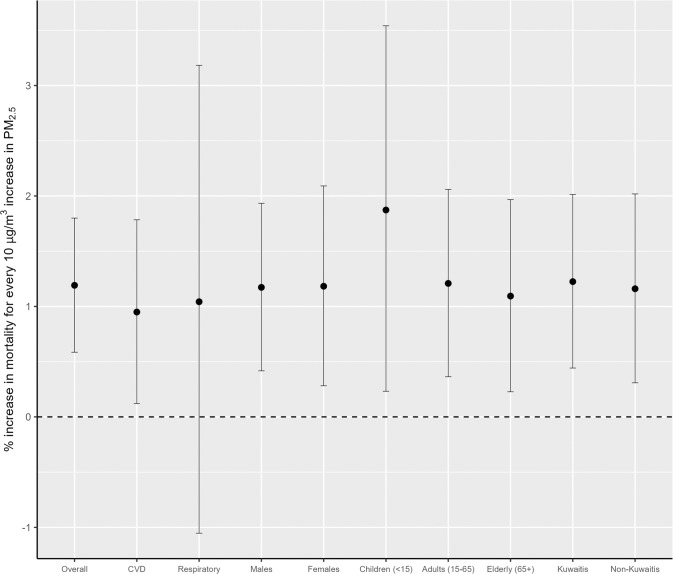
Percentage increase in mortality for every 10 µg/m^3^ increase in PM_2.5_ at 3-day moving average lag across different population strata in Kuwait.

All-causes and cardiovascular mortality were significantly associated with exposure to PM_2.5_, with a percent change of 1.19% (95% CI: 0.59, 1.80%) and 0.95% (95% CI: 0.12, 1.78%) per 10 µg/m^3^ increase in PM_2.5_ at lag03, respectively (Fig. 1). However, PM_2.5_ showed no significant association with respiratory mortality. For all-cause mortality, subgroup analyses were performed, and the analyses showed similar effects across most of the subgroups. were similar Specifically, the associations among males and females were 1.17% (95% CI: 0.42, 1.93%) and 1.18% (95% CI: 0.28, 2.09%), respectively. Across the three age groups, children showed a higher risk of dying from all-causes from exposure to PM_2.5_ (1.87%; 95% CI: 0.23, 3.54%), followed by adults (1.87%; 95%CI: 0.23, 3.54%) and the elderly (1.09%, 95% CI: 0.23, 1.97%). However, when comparing different risk estimates across groups, we found no statistically significant difference between Kuwaitis and non-Kuwaitis (*p* = 0.92), males and females (*p* = 0.94), as well as between adults and elderly people (*p* = 0.94).

**Fig. 2:**
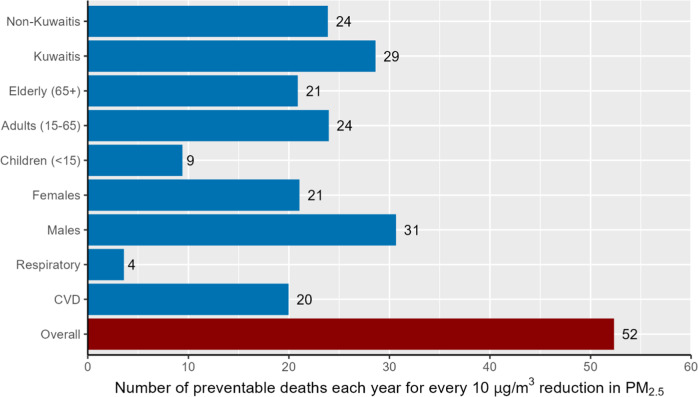
Number of potential yearly preventable deaths in Kuwait for every 10 µg/m^3^ reduction in annual PM_2.5_.

We estimated that 52.3 (95% CI: 25.7 to 79.1) deaths each year could be prevented had the average PM_2.5_ reduced by 10 µg/m^3^ in Kuwait (Fig. 2). That is, 28.6 (95% CI: 10.3 to 47.0) Kuwaitis and 23.9 (95% CI: 6.4 to 41.5) non-Kuwaitis each year. About 9.4 (95% CI: 1.2 to 17.8) and 20.9 (95% CI: 4.3 to 37.6) deaths in children and the elderly yearly may be preventable for a 10 µg/m^3^ reduction, respectively.

Further stratification of mortality risk and excess deaths is provided in the supplemental material (Table S1, Fig. S1).

## Discussion

In a region that is synonymous with yellow dusty skies, dust storms are getting more frequent, and policymakers are grappling with air pollution standards. We leveraged state-of-the-art estimates of spatiotemporally resolved PM_2.5_ to study air pollution vulnerability in Kuwait. A 10 µg/m^3^ increase in PM_2.5_ increases overall mortality by 1.19%. Should Kuwait’s PM_2.5_ concentrations reduce by 10 µg/m^3^, 52 lives could be saved each year; including approximately 9 children. Our models provide the essential foundations for a meaningful locally relevant air pollution risk assessment and risk management that can be applied successfully in desert climate regions with limited monitoring network.

The risk of mortality for a 10 µg/m^3^ increase in PM_2.5_ in Kuwait, where dust is the major component, was not too far off the global estimates. A study of 652 cities in 24 countries found that the global average increase in mortality per 10 µg/m^3^ increase in PM_2.5_ was 0.68% (95% CI: 0.59, 0.77%) [6]. While the global pooled effect estimate seems to be nearly half the one we found in Kuwait (0.68% vs. 1.19%), effect estimates from the US (1.58%), Canada (1.70%), Mexico (1.29%), Australia (1.42%), Spain (1.96%), Greece (2.54%), and Japan (1.42%) were all higher than the one we observed in Kuwait (1.19%). These differences may be attributable to a number of country-level factors (e.g., infrastructure, urbanization, ageing, distribution of co-morbidities, health care access, housing characteristics, and socio-economic resources). That said, there is, however, a great deal of uncertainty on whether the sources and composition of the particles themselves may be differentially toxic and vary by place of study [25]. There is some evidence, albeit inconclusive, that carbonaceous pollution (e.g., fossil-fuel burning, traffic emissions) may result in worse health effects compared to crustal dust [26]. In comparison to two similar smaller-size cities in the Eastern Mediterranean, which are also frequently affected dust storms, a 10 μg/m^3^ increase in PM_2.5_ resulted in 1.10% (−0.13, 2.34%) increase in cardiovascular mortality in Thessaloniki, and in 3.07% (−0.90, 7.20%) increase in all-cause mortality in Limassol [27]. A meta-analysis of all pollution-mortality studies in Iran found a pooled 1.5% (1.3, 1.7%) increase in all-cause mortality for a 10 μg/m^3^ increase in PM_2.5_ [28]. These estimates from the region are within the same range of Kuwait’s estimates seen in this paper. The evidence to date is yet to indicate a clear “hierarchy” of harmfulness for PM2.5 compositions.

Our findings suggest that PM_2.5_ is associated with increased mortality across nearly all strata of the Kuwaiti population with the risk of mortality for a 10 µg/m^3^ increase in PM_2.5_ being somewhat similar between subpopulations. This finding is particularly surprising on two fronts: age and nationality. First, we expected elders to be more vulnerable. The literature indicates that the elderly population is particularly vulnerable to air pollution and other environmental exposures [24, 29, 30]. One possible explanation could be an increased exposure to outdoor air pollution among adults, potentially resulting from outdoor work activities; however, we acknowledge that this is speculative and further research is needed to confirm this hypothesis. Second, the existing literature in Kuwait shows a clear health disparity between Kuwaitis and the non-Kuwaiti migrant workers [31–35]. For example, examining the effects of dust storm compared to non-dust storm days on mortality showed an effect measure modification by nationality in Kuwait where non-Kuwaitis were more vulnerable [15]. In our analysis, we found that both groups were equally vulnerable to PM_2.5_. The evidence, albeit inconclusive, is suggestive that non-Kuwaiti children were more vulnerable to air pollution compared to Kuwaiti children (stratification shown in the supplemental material Table S1, Fig. S1). More research is warranted to disentangle these health equity concerns.

In 2021, the World Health Organization (WHO) revised its PM_2.5_ annual guideline, reducing it from 10 μg/m^3^ to 5 μg/m^3^. The Kuwait Environmental Public Authority (KEPA) has yet to establish an annual PM_2.5_ standard. Notably, Kuwait’s annual PM_2.5_ concentrations are approximately at 46 μg/m^3^, far exceeding the WHO target. While these high annual concentrations are partly driven by frequent and extreme dust storms in Kuwait, recent research showed that anthropogenic contributions account for more than 50% of PM_2.5_ in the country [5]. Previous research suggested that there is a viable opportunity for regulators to reduce anthropogenic PM_2.5_ emissions, even if mitigating dust-related PM_2.5_ is considered infeasible [4, 36, 37].

Although our findings indicate that mortality effect estimates in other countries may be higher than those observed in Kuwait, it is important to consider the unique characteristics of Kuwait’s population and the differences in PM_2.5_ composition that may contribute to varying health risks. The implementation of air quality standards similar to those in US and Europe with larger mortality risks may provide a more comprehensive protection for the Kuwaiti population, as they are based on evidence of greater health risks. However, we acknowledge that current air quality standards are primarily based on PM_2.5_ concentration levels rather than composition. Further research is needed to better understand the factors driving differences in PM_2.5_ toxicity between Kuwait and other regions. This knowledge will help inform whether air quality regulations in Kuwait should be more or less strict than those in the US and Europe, and whether specific consideration should be given to the unique characteristics of Kuwait’s population and aerosols. In the meantime, efforts to reduce PM_2.5_ concentrations in Kuwait should continue, as they are likely to have substantial public health benefits.

This study has some limitations. First, we used predicted air pollution estimates from spatiotemporal models which are subject to exposure measurement error, despite a relatively good predictive ability (R^2^ = 0.71). Secondly, we were not able to obtain addresses from death certificates and therefore assumed that the population was uniformly exposed to the same daily concentration of PM_2.5_. While satellite-based PM2.5 predictions offer valuable insights for regions with limited ground monitoring data, it is important to note that aggregating these predictions over large spatial regions may not necessarily result in reduced exposure measurement errors compared to using information from a few monitoring stations alone. Nevertheless, the satellite-based predictions used in this study have enabled us to 1) assign exposure levels for Kuwait’s urban areas excluding barren desert lands, and 2) cover temporal gaps in PM2.5 measurements and conduct a comprehensive multi-decade time series analysis. Finally, the findings of this study are specific to the local context and population characteristics in Kuwait and may not be externally valid for other countries. The methods used in this study, however, should be extended to other similar desert regions where historical air pollution data does not exist.

## Conclusion

Studying air pollution epidemiology is exceedingly challenging in the Middle East and desert regions without a history of air quality monitoring. Nevertheless, as new tools are becoming available such as remote sensing, machine learning and retrospective prediction of PM_2.5_, scientists in the region are now able to characterize population health effects. We show that reducing 10 μg/m^3^ in PM_2.5_ in Kuwait could potentially save tens of lives each year. Countries in the Middle East need more local air pollution epidemiology studies to lay the ground for regional air quality standards.

## Supplementary information

Supplementary Information

Reporting Checklist

## Data Availability

The exposure data is available from the corresponding author upon reasonable request. Mortality data is subject to numerous restrictions by the source and could not be shared.
