# Supplementary material for: Burden of fine air pollution on mortality in the desert climate of Kuwait
Source: J Expo Sci Environ Epidemiol. 2023 Jun 15;33(4):646–51. doi: 10.1038/s41370-023-00565-7 (PMC10403355; doi:10.1038/s41370-023-00565-7)
Supplement: Supplementary file 1 — Supplementary Information [file 41370_2023_565_MOESM1_ESM.docx]

**
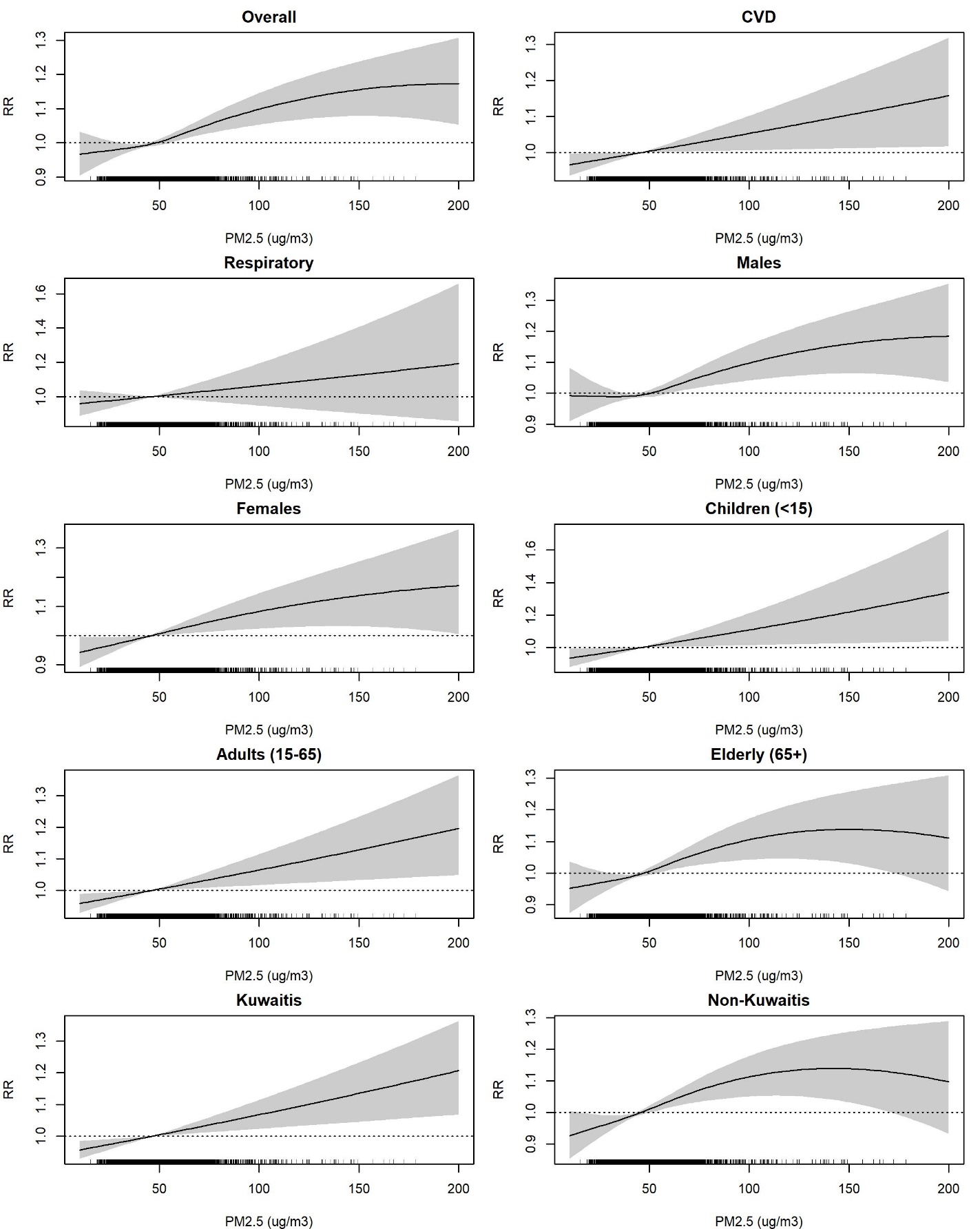
**

**Figure S1. Exposure-Response curves for mortality outcomes and 3-day moving average PM_2.5_ from generalized additive models (penalized splines). Models adjusted for time, day of the week, temperature and relative humidity.**

**Table S1. Deaths and PM_2.5_ risk estimates for different stratification of the Kuwaiti population. Results are from 3-day moving average timeseries quasipoisson models adjusted for time, day of the week, temperature and relative humidity from 2001 to 2016.**

| **Outcome** | **N** | **% of**  **deaths** | **Relative Risk for 10 ug/m^3^ increase** | | | **% increase in mortality for 10 ug/m^3^ increase** | | | **Number of preventable deaths for every 10 ug/m^3^ decrease** | | |
| --- | --- | --- | --- | --- | --- | --- | --- | --- | --- | --- | --- |
|  |  |  | *Est.* | *Lower* | *Upper* | *Est.* | *Lower* | *Upper* | *Est.* | *Lower* | *Upper* |
| Overall | 70321 | 100.0 | 1.012 | 1.006 | 1.018 | 1.19 | 0.59 | 1.80 | 52.3 | 25.7 | 79.1 |
| Kuwaitis | 37395 | 53.2 | 1.012 | 1.004 | 1.020 | 1.22 | 0.44 | 2.01 | 28.6 | 10.3 | 47.0 |
| Non-Kuwaitis | 32926 | 46.8 | 1.012 | 1.003 | 1.020 | 1.16 | 0.31 | 2.02 | 23.9 | 6.4 | 41.5 |
| Males | 41837 | 59.5 | 1.012 | 1.004 | 1.019 | 1.17 | 0.42 | 1.93 | 30.7 | 10.9 | 50.6 |
| Females | 28484 | 40.5 | 1.012 | 1.003 | 1.021 | 1.18 | 0.28 | 2.09 | 21.1 | 5.0 | 37.2 |
| CVD | 33649 | 47.9 | 1.009 | 1.001 | 1.018 | 0.95 | 0.12 | 1.78 | 20.0 | 2.5 | 37.5 |
| CVD Kuwaitis | 15612 | 22.2 | 1.016 | 1.004 | 1.027 | 1.56 | 0.42 | 2.71 | 15.2 | 4.1 | 26.4 |
| CVD Non-Kuwaitis | 18037 | 25.6 | 1.004 | 0.993 | 1.016 | 0.40 | -0.74 | 1.55 | 4.5 | -8.4 | 17.5 |
| CVD Males | 22681 | 32.3 | 1.008 | 0.998 | 1.018 | 0.83 | -0.17 | 1.85 | 11.8 | -2.4 | 26.2 |
| CVD Females | 10968 | 15.6 | 1.012 | 0.998 | 1.026 | 1.19 | -0.16 | 2.56 | 8.2 | -1.1 | 17.6 |
| Respiratory | 5512 | 7.8 | 1.010 | 0.989 | 1.032 | 1.04 | -1.05 | 3.18 | 3.6 | -3.6 | 11.0 |
| Respiratory Kuwaitis | 3581 | 5.1 | 1.013 | 0.988 | 1.039 | 1.30 | -1.23 | 3.89 | 2.9 | -2.8 | 8.7 |
| Respiratory Non-Kuwaitis | 1931 | 2.7 | 1.009 | 0.972 | 1.046 | 0.86 | -2.78 | 4.64 | 1.0 | -3.4 | 5.6 |
| Respiratory Males | 2910 | 4.1 | 1.025 | 0.996 | 1.055 | 2.52 | -0.37 | 5.48 | 4.6 | -0.7 | 10.0 |
| Respiratory Females | 2602 | 3.7 | 0.994 | 0.964 | 1.026 | -0.59 | -3.64 | 2.56 | -1.0 | -5.9 | 4.2 |
| Kuwaiti Males | 19832 | 28.2 | 1.015 | 1.004 | 1.025 | 1.45 | 0.40 | 2.52 | 18.0 | 4.9 | 31.2 |
| Kuwaiti Females | 17563 | 25.0 | 1.010 | 0.999 | 1.021 | 0.97 | -0.14 | 2.10 | 10.7 | -1.5 | 23.1 |
| Non-Kuwaiti Males | 22005 | 31.3 | 1.010 | 1.000 | 1.020 | 0.99 | -0.04 | 2.03 | 13.6 | -0.5 | 27.9 |
| Non-Kuwaiti Females | 10921 | 15.5 | 1.015 | 1.001 | 1.029 | 1.51 | 0.11 | 2.94 | 10.3 | 0.8 | 20.0 |
| Children (<15) | 8035 | 11.4 | 1.019 | 1.002 | 1.035 | 1.87 | 0.23 | 3.54 | 9.4 | 1.2 | 17.8 |
| Adults (15-65) | 31738 | 45.1 | 1.012 | 1.004 | 1.021 | 1.21 | 0.36 | 2.06 | 24.0 | 7.2 | 40.9 |
| Elderly (65+) | 30548 | 43.4 | 1.011 | 1.002 | 1.020 | 1.09 | 0.23 | 1.97 | 20.9 | 4.3 | 37.6 |
| Kuwaiti Children | 4525 | 6.4 | 1.012 | 0.990 | 1.035 | 1.24 | -0.95 | 3.48 | 3.5 | -2.7 | 9.8 |
| Non-Kuwaiti Children | 3510 | 5.0 | 1.028 | 1.004 | 1.053 | 2.81 | 0.41 | 5.26 | 6.2 | 0.9 | 11.5 |
| Kuwaiti Adults | 12252 | 17.4 | 1.017 | 1.004 | 1.030 | 1.73 | 0.43 | 3.04 | 13.2 | 3.3 | 23.3 |
| Non-Kuwaiti Adults | 19486 | 27.7 | 1.008 | 0.997 | 1.019 | 0.79 | -0.30 | 1.89 | 9.6 | -3.7 | 23.1 |
| Kuwaiti Elderly | 20618 | 29.3 | 1.010 | 0.999 | 1.020 | 0.96 | -0.08 | 2.00 | 12.3 | -1.0 | 25.8 |
| Non-Kuwaiti Elderly | 9930 | 14.1 | 1.014 | 0.999 | 1.029 | 1.39 | -0.10 | 2.90 | 8.6 | -0.6 | 18.0 |


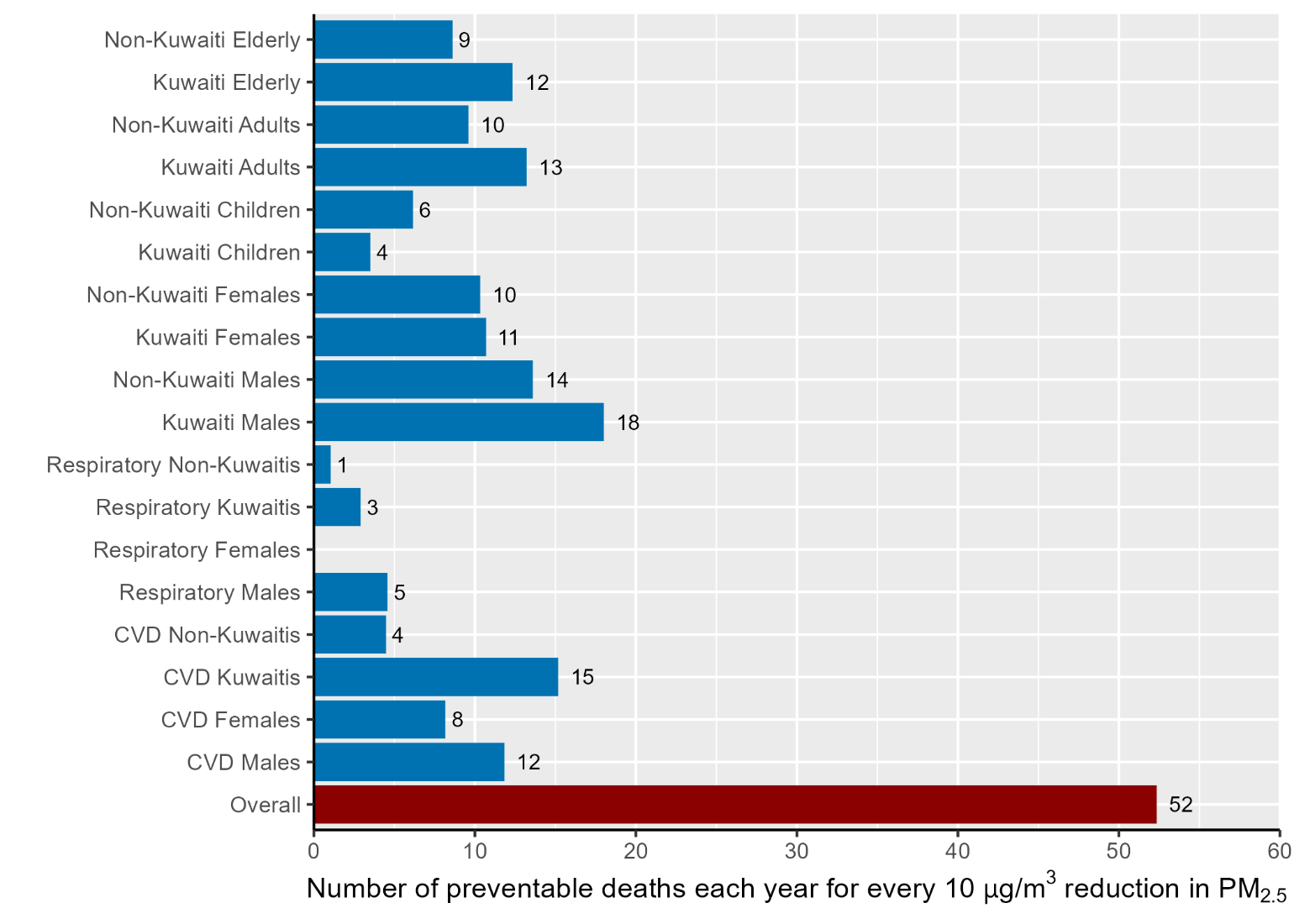


**Figure S2. Number of potential yearly preventable deaths in Kuwait for every 10 µg/m^3^ reduction in PM_2.5_ among stratified subgroups of the Kuwaiti population.**
